# Supplementary material for: WordCommentsAnalyzer: A windows software tool for qualitative research
Source: F1000Res. 2018 Sep 4;7:536. Originally published 2018 May 3. [Version 2] doi: 10.12688/f1000research.14819.2 (PMC6137411; doi:10.12688/f1000research.14819.2)
Supplement: Supplementary file 3 [file f1000research-7-17614-s0002.tgz › b2a79496-4ada-47c4-9682-a99ce867836d.zip!/Eight Iranian Health Journals/Salmand_Iranian Journal of Ageing _3.docx]

© Salmand_Iranian Journal of Ageing . This work is licensed under CC BY-NC 4.0 (https://creativecommons.org/licenses/by-nc/4.0/).

Volume & Issue: دوره 12 - شماره 3

Title: مقایسه کیفیت زندگی زنان سالمند فعال و غیرفعال با تأکید بر فعالیت‌های بدنی

http://salmandj.uswr.ac.ir/article-1-1247-fa.html

اهداف مقوله کیفیت زندگی در قشرهای مختلف، مخصوصاً سالمندان که شرایط جسمی، روحی و روانی ویژه‌ای دارند اهمیت زیادی دارد. هدف از تحقیق حاضر مقایسه کیفیت زندگی بین زنان سالمند فعال و غیرفعال شهرستان بندرگز با تأکید بر فعالیت‌های بدنی بود. مواد و روش ها پژوهش حاضر از نوع تحقیقات توصیفی‌پیمایشی است. جامعه آماری این تحقیق تمام سالمندان زن بالای ۶۰ سال شهرستان بندرگز بودند. نمونه‌گیری به صورت در دسترس انجام شد و تعداد نمونه‌های تحقیق ۱۷۶ نفر بود. به منظور گردآوری داده‌های این تحقیق از پرسش‌نامه SF-۳۶ استفاده شد. به منظور تجزیه‌وتحلیل آماری داده‌ها از آزمون کلموگروف اسمیرنوف و آزمون یومن ویتنی استفاده شد. تمام عملیات آماری با استفاده از نرم‌افزار SPSS نسخه ۲۰ در سطح معناداری P≤۰/۰۵ انجام شد. یافته ها با توجه به نتایج این تحقیق، میانگین سن گروه فعال ۵/۵۴±۶۵/۶۹ و گروه غیرفعال ۵/۱۴±۶۴/۳۲ بود. زنان فعال نسبت به زنان غیرفعال در شاخص‌های محدودیت عملکرد بدنی به ترتیب نمره ۱۷/۲±۴۹/۳ و ۱۱/۳±۶۶/۲، مشکلات جسمانی ۱۴/۸±۴۵/۹ و ۱۲/۴±۶۲/۸، مشکلات عاطفی ۱۶/۶±۳۱/۵ و ۱۴/۱±۵۰/۴، انرژی و سرزندگی ۱۴/۷±۵۹/۷ و ۱۱/۵±۵۲/۲، سلامت روانی ۱۷/۹±۵۶/۶ و ۱۴/۲±۴۵/۳، درد ۱۲/۳±۴۱/۲ و ۱۷/۷±۵۸/۳ و سلامت عمومی ۱۳/۹±۶۰/۱ و ۱۵/۴±۴۹/۳ را کسب کردند و کیفیت زندگی بالاتری داشتند، ولی در شاخص عملکرد اجتماعی تفاوت معناداری مشاهده نشد ۱۷/۶±۵۰/۹ در مقابل ۱۶/۲±۴۷/۳. سلامت روانی و سلامت عمومی به عنوان مهم‌ترین ابعاد کیفیت زندگی سالمندان شناخته شد.  نتیجه گیری با توجه به افرایش تعداد سالمندان، توجه به ابعاد مختلف کیفیت زندگی آنان اهمیت فراوانی دارد. پرداختن به فعالیت‌های بدنی منظم می‌تواند به عنوان یکی از راهکارهای بهبود وضعیت کیفیت زندگی سالمندان مورد توجه قرار گیرد. پیشنهاد می‌شود مسئولان و دست‌اندرکاران امر سلامت، ایجاد امکانات مناسب برای ورزش و فعالیت بدنی سالمندان را مدنظر قرار دهند.

Title: ارتباط بین تفکر قطعی‌نگر با احساس تنهایی و افسردگی در سالمندان

http://salmandj.uswr.ac.ir/article-1-1076-fa.html

اهداف تفکر قطعی‌نگر به عنوان عامل مخربی در مختل‌کردن تعادل بین امید و ترس نقش مهمی در سلامت روان به خصوص افسردگی و اضطراب دارد. این تحریف در اثر انعطاف‌ناپذیری شناختی در ذهن ظاهر می‌شود، به طوری که خود یکی از دلایل مهم افسردگی و دیگر ناسازگاری‌های روانی است. این مطالعه با هدف بررسی ارتباط بین تفکر قطعی‌نگر با افسردگی و احساس تنهایی در سالمندان انجام گرفت.  مواد و روش ها مطالعه حاضر از نوع توصیفی‌همبستگی بود. جامعه آماری شامل تمامی سالمندان بیش از ۶۰ سال ساکن در آسایشگاه سالمندان کرج در سال ۹۴-۹۳ بود که از این بین ۱۴۲ سالمند (زن و مرد) به روش نمونه‌گیری در دسترس انتخاب و به پرسش‌نامه‌های تفکر قطعی‌نگر (DTQ) مقیاس افسردگی سالمندان (GDS)، مقیاس احساس تنهایی راسل (UCLA) پاسخ دادند. داده‌های گردآوری‌شده برای تجزیه‌وتحلیل وارد نرم‌افزار AMOS و با استفاده از مدل تحلیل مسیر، تحلیل شدند. یافته ها نتایج تحلیل مسیر نشان داد که تفکر قطعی‌نگر با متغیرهای افسردگی (P≤۰/۰۰۱) و احساس تنهایی (P≤۰/۰۰۱) رابطه مثبت معنی‌داری داشت. همچنین در پیش‌بینی احساس تنهایی با میزان اتای ۰/۲۶ و در پیش‌بینی افسردگی با میزان اتای ۰/۲۸ ضریب تأثیر معنی‌داری داشت. میانگین سنی زنان سالمند ۶۷/۲ و مردان سالمند ۶۵/۴ و نیز میانگین متغیرهای تفکر قطعی‌نگر، احساس تنهایی و افسردگی در دو گروه زنان و مردان به ترتیب ۱۱۸/۵۰، ۷۰/۸۰ و ۱۲/۵۵ است.  نتیجه گیری با توجه به نتایج به‌دست‌آمده می‌توان گفت که تفکر قطعی‌نگر با افسردگی و احساس تنهایی در سالمندان رابطه معناداری دارد. همچنین تفکر قطعی‌نگر پیش‌بینی‌کننده افسردگی و احساس تنهایی در سالمندان است. بنابراین انجام مداخلات روان‌شناختی برای به چالش‌کشاندن تحریف شناختی تفکر قطعی‌نگر و توجه به سلامت روان سالمندان بسیار حائز اهمیت است.

Title: تأثیر مداخلات نوروفیدبک و تصویرسازی ذهنی بر تعادل سالمندان

http://salmandj.uswr.ac.ir/article-1-1105-fa.html

اهداف تعادل یکی از شاخص‌های تعیین استقلال افراد سالمند به شمار می‌رود. بررسی و تشخیص عوامل مؤثر بر آن از موضوعاتی است که محققان به آن توجه کرده‌اند. هدف از انجام مطالعه حاضر بررسی اثر تمرینات نوروفیدبک و تصویرسازی حرکتی بر تعادل سالمندان است.  مواد و روش ها جامعه آماری مدنظر سالمندان شهر مشهد بودند که از بین آن‌ها ۲۴ سالمند در دامنه سنی ۶۰ تا ۸۲ سال به‌صورت داوطلبانه انتخاب و سپس به سه گروه ۸ نفری به‌صورت تصادفی تقسیم شدند. شرکت‌کنندگان دو گروه تجربی به مدت ۸ جلسه (۳ جلسه در هفته) در تمرینات مربوط به خود (تمرین نوروفیدبک و تمرین تصویرسازی حرکتی) شرکت کردند، اما گروه کنترل هیچ‌گونه تمرینی انجام نداد. در این تحقیق برای اندازه‌گیری تعادل ایستای سالمندان از آزمون تعادلی لک‌لک و برای اندازه‌گیری تعادل پویای آن‌ها از آزمون زمان برخاستن و راه رفتن، استفاده شد که در دو مرحله قبل و بعد از تمرینات ارزیابی می‌شدند. به‌منظور تعیین عادی‌بودن توزیع داده‌ها از آزمون شاپیرو ویلکس و برای تعیین آثار مرتبط با تمرین از تحلیل کوواریانس استفاده شد. نمرات پیش‌آزمون افراد به‌عنوان متغیر مداخله‌گر مدنظر قرار گرفت. تمامی آزمون‌های آماری با نرمافزار SPSS نسخه ۲۱ و در سطح معنی‌داری ۰/۰۵=a تحلیل شدند. یافته ها نتایج حاصل از آزمون تحلیل کوواریانس نشان‌دهنده تأثیر معنی‌دار تمرینات نوروفیدبک و تصویرسازی حرکتی بر تعادل ایستا و پویای سالمندان بود (۰/۰۵>P). ضمن اینکه تمرین نوروفیدبک بر هر دو وضعیت تعادل ایستا و پویا تأثیر بهتری داشت (۰/۰۵>P).  نتیجه گیری با توجه به نتایج به‌دست‌آمده پیشنهاد می‌شود از این تمرینات به‌منظور پیشگیری از افت تعادل و تقویت آن در سالمندان استفاده شود.

Title: رابطه تاب‌آوری، خودکارآمدی و حمایت اجتماعی با رضایت شغلی در بین میان‌سالان و سالمندان شاغل

http://salmandj.uswr.ac.ir/article-1-1289-fa.html

اهداف با توجه به اهمیت موضوع رضایت شغلی در بین میان‌سالان و سالمندان، پژوهش حاضر با هدف بررسی رابطه تاب‌آوری، خودکارآمدی و حمایت اجتماعی با رضایت شغلی در بین میان‌سالان و سالمندان شاغل شهر کرمانشاه انجام شد. مواد و روش ها جامعه آماری پژوهش شامل تمامی میان‌سالان و سالمندان شاغل شهر کرمانشاه در سال ۱۳۹۵ بود که نمونه‌ای به حجم ۲۴۰ نفر به روش نمونه‌گیری در دسترس از بین آن‌ها انتخاب شد؛ شامل۱۶۰ نفر مرد و ۸۰ نفر زن در دامنه سنی بین ۵۰ تا ۷۵ سال. میانگین سنی آن‌ها نیز ۱/۹۳±۶۶/۸۷ بود. برای گردآوری داده‌های پژوهش از پرسش‌نامه‌های باورهای خودکارآمدی عمومی (GSE-۱۰)، پرسش‌نامه کونورو دیویدسون (CD-RIS)، پرسش‌نامه رضایت شغلی اسپکتور (JSS) و پرسش‌نامه مقیاس ادراک حمایت اجتماعی چندبعدی استفاده شد.  یافته ها تجزیه‌وتحلیل داده‌ها با استفاده از ضریب همبستگی و تحلیل رگرسیون انجام شد. نتایج نشان داد بین خودکارآمدی و تاب‌آوری با رضایت شغلی رابطه مثبت وجود دارد. همچنین بین حمایت اجتماعی با رضایت شغلی میان‌سالان و سالمندان شاغل نیز رابطه مثبت و معناداری وجود دارد.  نتیجه گیری با توجه به یافته‌های این مطالعه می‌توان نتیجه گرفت که خودکارآمدی، تاب‌آوری و حمایت اجتماعی به عنوان متغیرهای پیش‌بین توان پیش‌بینی رضایت شغلی میان‌سالان و سالمندان شاغل را دارند.

Title: اثربخشی درمان گروهی مرور زندگی بر بهزیستی روان‌شناختی و امید به زندگی زنان سالمند

http://salmandj.uswr.ac.ir/article-1-1152-fa.html

اهداف سالمندی دوران حساسی از زندگی بشر است. با افزایش جمعیت سالمندان، توجه به مسائل و نیازهای این مرحله، خصوصاً سالمندان ساکن آسایشگاه‌ها ضروری به نظر می‌رسد. پژوهش حاضر با هدف بررسی اثربخشی درمان گروهی مرور زندگی بر بهزیستی روان‌شناختی و امید به زندگی زنان سالمند ساکن شهر اردبیل صورت گرفت. مواد و روش ها مطالعه حاضر نیمه‌آزمایشی، با طرح پیش‌آزمون پس‌آزمون با گروه کنترل بود. جامعه آماری پژوهش را تمامی زنان سالمند ساکن آسایشگاه‌های شهر اردبیل در سال ۱۳۹۵ تشکیل می‌دادند. به منظور اجرای این مطالعه، ۳۰ نفر از زنان ساکن این آسایشگاه‌ها که موقعیت ورود به مطالعه را داشتند، به‌طور تصادفی ساده انتخاب شدند و به طور مساوی در دو گروه مداخله (۱۵ نفر) و گواه (۱۵ نفر) قرار گرفتند. برای گردآوری داده‌ها از مقیاس‌های بهزیستی روان‌شناختی ریف (۱۹۸۹) و امید اشنایدر (۱۹۹۱) استفاده شد. پس از اجرای پیش‌آزمون، گروه‌درمانی مرور زندگی با تأکید بر نظریه اریکسون، به صورت شش جلسه گروهی و به مدت ۶ هفته و ۱ جلسه (۹۰ دقیقه‌ای) در هفته برای گروه مداخله اجرا شد. برای گروه گواه، هیچ مداخله درمانی صورت نگرفت و در فهرست انتظار ماندند. پس از پایان آموزش، پس‌آزمون برای هر دو گروه تکرار شد. داده‌های حاصل، با استفاده از آزمون تحلیل کوواریانس چندمتغیره و به کمک نرم‌افزار spss نسخه ۲۰ تجزیه‌وتحلیل شدند. یافته ها نتایج مطالعه حاضر نشان داد که میانگین نمرات مؤلفه‌های خودمختاری، تسلط بر محیط، رشد شخصی، روابط مثبت با دیگری، هدف در زندگی و پذیرش خود بین گروه‌های مداخله و گواه تفاوت معناداری وجود دارد (P<۰/۰۱). همچنین نتایج نشان داد که این درمان باعث افزایش امید به زندگی زنان سالمند در گروه مداخله شده است (P<۰/۰۵). نتیجه گیری درمان گروهی مرور زندگی، افزایش بهزیستی روان‌شناختی و امید به زندگی در سالمندان را به دنبال دارد. از این رو نظر به اهمیت مسئله سالمندی و مسائل روان‌شناختی آن و با توجه به نتایج این مطالعه، ‌می‌توان از این روش درمانی به‌عنوان یکی از درمان‌های حمایتی و مؤثر روان‌شناسان و پزشکان شاغل در حوزه‌ سالمند بهره جست.

Title: بررسی رابطه طرح‌واره‌های ناسازگار اولیه با معنای زندگی و بهزیستی روان‌شناختی سالمندان

http://salmandj.uswr.ac.ir/article-1-1320-fa.html

اهداف هدف از پژوهش حاضر بررسی رابطه طرح‌واره‌های ناسازگار اولیه با معنای زندگی و بهزیستی روان‌شناختی سالمندان است.  مواد و روش ها به منظور انجام پژوهش، ۲۰۰ نفر (۹۶ زن و ۱۰۴ مرد) از سالمندان شهر تهران در سال ۱۳۹۵ از طریق نمونه‌گیری در دسترس انتخاب شدند و با استفاده از پرسش‌نامه‌های طرح‌واره یانگ، معنای زندگی استگر و بهزیستی روان‌شناختی ریف بررسی شدند.  یافته ها نتایج تحلیل رگرسیون چندمتغیره هم‌زمان نشان داد ۳۷ درصد از جست‌وجوی معنا، ۲۷ درصد از وجود معنا، ۲۷ درصد از پذیرش خود، ۲۴ درصد از روابط مثبت با دیگران و ۴۴ درصد از تسلط بر محیط به‌وسیله طرح‌واره‌های ناسازگار اولیه قابل پیش‌بینی است. خرده‌مقیاس‌های رشد فردی، زندگی هدفمند و خودمختاری قابلیت پیش‌بینی به‌وسیله طرح‌واره‌ها را ندارند. این همبستگی از نظر آماری معنی‌دار است (P<۰/۰۰۱). مدل رگرسیونی تبیین‌شده نیز بر اساس آزمون تحلیل واریانس، خطی و معنی‌دار است، زیرا مقدار آزمون f برای تعیین معنی‌داری اثر متغیرهای مستقل بر جست‌وجوی معنا و وجود معنا ۲۱/۵ و ۲۲/۳ با سطح معنی‌داری P<۰/۰۰۱ است و برای اثر متغیرهای مستقل بر مؤلفه‌های بهزیستی روان‌شناختی پذیرش خود ۲۳/۳=f، روابط مثبت با دیگران۶۸/۲=f، تسلط بر محیط ۱۷/۲=f، سطح معنی‌داری P<۰/۰۰۱ گزارش شد.  نتیجه گیری نتایج حاصل از این پژوهش نشان داد طرح‌واره‌های ناسازگار اولیه معیارهای سخت‌گیرانه، ایثار، اطاعت، محرومیت هیجانی، انزوای اجتماعی و شکست، توانایی پیش‌بینی‌کنندگی معنای زندگی و بهزیستی روان‌شناختی را دارند و بر سلامت روان در دوران سالمندی با میانگین سنی ۶۵ تا ۸۰ سال تأثیرگذار هستند.

Title: بررسی رابطه عوامل ادراکی و شاخصه‌های اجتماعی در طراحی اقامتگاه‌های سالمندی شفابخش

http://salmandj.uswr.ac.ir/article-1-1242-fa.html

اهداف پدیده‌ سالمندی جمعیت در جهان و ایران با توجه به آمار مستند، موضوعی انکارناپذیر است. از نیازهای مهم سالمندان پاسخ‌گویی به دو مشکل ضعف ادراک و انزوای اجتماعی است. ازاین‌رو ضروری است که محیط مناسب و بهتری برای زندگی آن‌ها فراهم شود تا افسردگی‌های ناشی از تنهایی کاهش یابد. هدف پژوهش حاضر بررسی اهمیت دو عامل ادراکی و اجتماعی و رابطه میان این دو متغیر با توجه به ویژگی‌های شفابخشی محوطه خارجی اقامتگاه سالمندان است.  مواد و روش ها پژوهش حاضر طی روندی پیمایشی، با بررسی ۱۴۸ سالمند (۷۵ مرد و ۷۳ زن) مقیم در ۹ اقامتگاه سالمندان تهران و ارائه‌  پرسش‌نامه‌ محقق ساخت (آلفای کرونباخ معادل ۰/۹۰۴) به بررسی رابطه‌ عوامل ادراکی (روانی و عاطفی) و اجتماعی پرداخته است.  یافته ها روابط متغیرهای پژوهش با اطلاعات جمعیت‌شناختی شرکت‌کنندگان تحلیل شد. میانگین سنی سالمندان شرکت‌کننده در پژوهش برابر ۸۳ سال و انحراف معیار آن برابر ۰/۵۸ به دست آمد. تحلیل‌ها توسط آمار توصیفی و استنباطی (همبستگی پیرسون با ضریب اطمینان ۰/۹۹ و سطح معنی‌داری P≤۰/۰۵ و تحلیل واریانس LSD فیشر) در محیط نرم‌افزار SPSS۲۲ صورت پذیرفت. یافته­‌ها بیان­گر رابطه بین عوامل ادراکی‌روانی (P=۰/۰۰۱) و اجتماعی‌فرهنگی (P=۰/۰۰۱) با عامل ادراکی‌عاطفی و رابطه بین عوامل ادراکی‌عاطفی (P=۰/۰۰۱) و اجتماعی‌فرهنگی (P=۰/۰۰۳) با عامل ادراکی‌روانی است. همچنین یافته‌ها نشان‌دهنده­ وجود روابط معنی‌دار در مشخصات جمعیت‌شناختی سن، مدت اقامت در مرکز، وضعیت تأهل و علت رجوع به مرکز، در گروه‌های سالمندان با یکدیگر بود. نتیجه گیری بر اساس یافته‌ها چنانچه عوامل ادراکی (عاطفی و روانی) شفابخش محیط سالمندان دچار تغییر شود، روابط اجتماعی و فرهنگی او می‌تواند مستقیم تحت تأثیر قرار گیرد. باتوجه به مشخصات جمعیت‌شناختی نیز می‌توان نتیجه گرفت برخی عوامل به دلیل برخی ویژگی‌های افراد، تحت تأثیر قرار می‌گیرند. می­‌توان این مشخصات را در معماری لحاظ کرد و زمینه‌های رشد کیفیت زندگی سالمندان را فراهم آورد.

Title: بررسی آسیب‌پذیری اجتماعی سالمندان در زلزله‌های بم، ورزقان و اهر

http://salmandj.uswr.ac.ir/article-1-1239-fa.html

اهداف زلزله حادثه‌ای طبیعی است، اما به سبب آثار اجتماعی گوناگونی که دارد به موضوعی جامعه‌شناختی تبدیل شده است و گروه‌های آسیب‌پذیری را به وجود می‌آورد. یکی از این گروه‌ها سالمندان هستند. هدف این پژوهش بررسی آسیب‌پذیری اجتماعی سالمندان در زلزله بود. مواد و روش ها پژوهش حاضر از نوع کیفی‌کمی بود که به‌ صورت مقطعی در سال ۱۳۹۴ انجام شد. جامعه آماری شامل سالمندان استان‌های آذربایجان شرقی و کرمان بود. در بخش کیفی به‌منظور شناخت آسیب‌های اجتماعی سالمندانی که زلزله را تجربه کرده‌اند، از طرح تحقیق پدیدارشناسی با بهره‌گیری از گروه‌های کانونی استفاده شد. بدین منظور هفده نفر به روش نمونه‌گیری هدفمند برای گروه کانونی سالمندان انتخاب و آسیب‌های اجتماعی زلزله، شناسایی شدند. در مرحله کمی، نود نفر از سالمندانی که زلزله‌های بم، ورزقان و اهر را تجربه کرده بودند، انتخاب شدند و سپس نود نفر که زلزله را تجربه نکرده بودند، به‌طور همتا با گروه اول انتخاب‌شدند. سپس به سؤالات پرسش‌نامه انزوای اجتماعی، پرسش‌نامه حمایت اجتماعی، پرسش‌نامه سازگاری اجتماعی SAS پی کل و ویسمن و مقیاس پذیرش اجتماعی مارلوکراون پاسخ دادند. دادهای گردآوری شده با نسخه ۲۱ نرم‌افزار SPSS و آزمون T تجزیه‌وتحلیل شدند. یافته ها در بخش کیفی، آسیب‌های اجتماعی زلزله شامل ناسازگار ی اجتماعی، انزوای اجتماعی، عدم‌پذیرش اجتماعی و عدم‌حمایت اجتماعی از طریق مصاحبه‌ها شناسایی شدند. در بخش کمی نتایج نشان داد میانگین ناسازگاری اجتماعی سالمندان حاضر در زلزله (۰/۶۶±۴/۹۳) و سالمندان دیگر (۱/۱۶±۳/۴۲)، میانگین انزوای اجتماعی سالمندان حاضر در زلزله (۳/۹۱±۱۲/۲۳) و سالمندان دیگر (۳/۱۷±۸/۰۶)، میانگین پذیرش اجتماعی سالمندان حاضر در زلزله (۲/۳۸±۱۱/۴۱) و سالمندان دیگر (۴/۶۶±۲۴/۷) و میانگین حمایت اجتماعی سالمندان حاضر در زلزله (۶/۸۱±۳۴/۱۲) و سالمندان دیگر (۸/۹۶±۶۸/۹) بود. بنابراین میزان سازگاری اجتماعی سالمندان زلزله‌زده کمتر و انزوای اجتماعی آنان بیشتر از سالمندان دیگر بود (۰/۰۱>P). همچنین یافته‌ها نشان داد که احساس حمایت و پذیرش اجتماعی سالمندان زلزله‌زده کمتر از سالمندان دیگر بود (۰/۰۱>P). نتیجه گیری بر اساس یافته‌های مطالعه حاضر، سالمندان از جمله گروه‌های اجتماعی آسیب‌پذیر در زلزله هستند که علاوه بر اختلال حافظه و ازدست‌دادن جهت‌یابی و آسیب‌های روانی، به آسیب‌های اجتماعی نیز دچار خواهد شد. به این آسیب‌ها کمتر توجه شده است. بنابراین برنامه‌ریزی و اتخاذ تدابیر لازم برای کاهش و کنترل این‌گونه آسیب‌ها، یکی از مهم‌ترین ضرورت‌های پیش رو خواهد بود.

Title: همه‌گیرشناسی تروما در میان مددجویان سالمند امدادرسانی‌شده در سیستم اورژانس پیش‌بیمارستانی مازندران طی سال‌های1389 تا 1393

http://salmandj.uswr.ac.ir/article-1-1244-fa.html

اهداف در حال حاضر جهان به‌سرعت به سمت سالمندشدن پیش می‌رود؛ کشور ایران نیز از این امر مستثنا نیست. تروما پنجمین علت مرگ در بیماران بالای ۶۵ سال است و ۲۸ درصد از این افراد بر اثر تروما فوت می‌کنند. به منظور تدوین راهکارهای پیشگیری از بروز صدمات در این گروه سنی پرخطر پژوهش حاضر با هدف تعیین الگوی بروز تروما در افراد ۶۰ سال و بالاتر به تفکیک سن و جنس انجام شده است. مواد و روش ها در این مطالعه مقطعی گذشته‌نگر، از اطلاعات مربوط به تمام افراد مسن ۶۰ سال و بالاتر پذیرفته‌شده در اورژانس پیش‌بیمارستانی مازندران طی سال‌های ۱۳۸۹ تا ۱۳۹۳، مواردی چون سن، جنس، تشخیص پیش‌بیمارستانی، تاریخ، نوع صدمه، مکانیسم بروز تروما، محل بروز حادثه، وضعیت مصدوم (راننده اتومبیل، سرنشین، عابر و غیره)، شدت جراحت (جراحت سطحی، شدید و نقص عضو)، فصل و زمان وقوع حادثه و غیره استخراج شد. به منظور تحلیل آماری داده‌ها از نرم‌افزار SPSS نسخه ۱۹ و آزمون دقیق فیشر و کای دو پیرسون استفاده شد. یافته ها میزان بروز تروما در سال ۱۹۳ نسبت به سال ۱۳۸۹، ۱/۷ درصد افزایش داشته است. ۶هزار و ۸۴۴ نفر (۶۲/۸ درصد) از افراد بررسی‌شده مرد و ۴هزار و ۵۴ نفر (۳۷/۲ درصد) زن بودند. بیشترین میزان تروما از لحاظ سنی با ۳هزار و ۶۸۰ نفر (۳۳/۸ درصد) در گروه سنی ۶۰ تا ۶۴ سال قرار داشتند. بیشترین موارد تروما در سالمندان مربوط به حوادث ترافیکی به تعداد ۶هزار و ۵۲۱ (۵۹/۸ درصد) بود. پس از تصادف، سقوط، صدمات و ضرب‌وجرح به ترتیب با ۳هزار و ۴۹۴ (۳۲ درصد)، ۵۱۷ (۴/۷ درصد) و ۲۵۷ (۲/۴ درصد) بیشترین موارد را به خود اختصاص داده‌اند. بیشترین فصل بروز تروما با ۲هزار و ۹۸۲ مورد (۲۷/۴ درصد) در تابستان و بیشترین زمان بروز تروما در ساعات ۸ صبح تا ۱۲ ظهر به تعداد ۳هزار و ۱۱۹ مورد (۲۸/۶ درصد) بوده است. با توجه به آزمون کای دو پیرسون بین وضعیت افراد هنگام تصادف و وضعیت نهایی رابطه آماری معنی‌داری وجود دارد (P<۰/۰۵). بیشترین زمان بروز فوت نیز در گروه سنی ۶۰ تا ۶۴ سال بوده است. شانس زنده‌ماندن در مردان نسبت به زنان کمتر است. نتیجه گیری سالمندان به علت تغییرات جسمی و حرکتی ناشی از افزایش سن و نامناسب‌بودن شرایط محیط بیشتر در معرض حوادث قرار می‌گیرند. طبق نتایج این پژوهش، تصادفات و سقوط از شایع‌ترین حوادث در دوره سالمندی است. با برنامه‌ریزی‌های جامع در زمینه کنترل و پیشگیری می‌توان از بروز آسیب و ناتوانی در این گروه سنی پرخطر پیشگیری کرد.

Title: تأثیر هشت هفته تمرین‌های آبی قسمت عمیق استخر بر تعادل ایستای مردان سالمند

http://salmandj.uswr.ac.ir/article-1-1305-fa.html

اهداف در این تحقیق ۳۰ مرد سالمند به طور داوطلبانه و به شکل تصادفی در دو گروه ۱۵ نفری کنترل و تجربی شرکت کردند. گروه تجربی در برنامه تمرین‌های آبی در قسمت عمیق استخر به مدت هشت هفته که شامل سه جلسه یک ساعته در هفته بود شرکت کردند؛ درحالی‌که گروه کنترل در این مدت در هیچ برنامه تمرینی شرکت نکردند. در هر دو گروه تعادل ایستا قبل و بعد از دوره اندازه‌گیری شد. شاخص‌های نوسانات پاسچر (تعادل ایستا) با استفاده از دستگاه تعادلی بایودکس و آزمون ثبات پاسچرال اندازه‌گیری شد. برای مقایسه تغییرات درون‌گروهی از آزمون تی همبسته و برای مقایسه شاخص‌های تعادل بین دو گروه از آزمون تی مستقل استفاده شد. مواد و روش ها ۳۰ مرد سالمند، به طور داوطلبانه و به شکل تصادفی در دو گروه ۱۵ نفره کنترل و تجربی در این تحقیق شرکت کردند. گروه تجربی در برنامه تمرینات آبی در قسمت عمیق استخر به مدت هشت هفته که شامل سه جلسه یک ساعته در هفته بود شرکت کرد. این در حالی بود که گروه کنترل در این مدت در هیچ گونه برنامه تمرینی شرکت نکردند. در هر دو گروه تعادل ایستا قبل و بعد از دوره اندازه گیری شد. شاخص های نوسانات پاسچر (تعادل ایستا) با استفاده از دستگاه تعادلی بایودکس و تست ثبات پاسچرل اندازه‌گیری شد. از آزمون تی همبسته برای مقایسه تغییرات درون گروهی و از تست تی مستقل برای مقایسه شاخص های تعادل بین دو گروه استفاده شد. یافته ها نتایج مربوط به مقایسه دو گروه از نظر ویژگی‌های جمعیت‌شناختی و تن‌سنجی نشان داد گروه تجربی میانگین سن ۳/۴۸۱±۶۵/۵۰، قد ۵/۲۱۵±۱۶۸/۱۰، جرم ۷/۷۷۵±۷۰/۸۷ و شاخص توده بدنی۲/۴۸۳±۲۴/۷۸۱ و گروه کنترل میانگین سن۳/۲۳۲±۶۶/۵۰، قد ۶/۳۱۴±۱۶۹/۲۰، جرم ۸/۵۳۱±۷۱/۳۲۲ و شاخص توده بدنی ۳/۵۳۵±۲۴/۹۸۲ دارند. این نتایج حاکی از نبود تفاوت و درنتیجه همگنی دو گروه است. همچنین نتایج نشان داد هشت هفته تمرین‌های آبی در قسمت عمیق استخر، باعث بهبود محسوسی در تعادل ایستا آزمودنی‌ها شده است. نتایج آزمون‌های تی مستقل و همبسته نشان‌دهنده تفاوت معناداری بین گروه تجربی وکنترل در پس‌آزمون و در گروه تجربی بین پیش‌آزمون و پس‌آزمون بعد از انجام برنامه هشت هفته‌ای است. نتیجه گیری با توجه به یافته‌های تحقیق می‌توان گفت که اعمال برنامه آبی در قسمت عمیق استخر می‌تواند در بهبود تعادل ایستا و درنهایت کاهش خطر افتادن در سالمندان مؤثر باشد.
